# Cardiac CT reveals high prevalence of coronary artery disease in esophageal cancer eligible for radiotherapy

**DOI:** 10.2340/1651-226X.2025.42563

**Published:** 2025-02-03

**Authors:** Akinori Takada, Satoshi Nakamura, Yutaka Toyomasu, Takamitsu Mase, Tomoko Kawamura, Suguru Araki, Yoshitaka Suzuki, Masaki Ohi, Hajime Sakuma, Yoshihito Nomoto, Kakuya Kitagawa

**Affiliations:** a Department of Radiology, Mie University Hospital, Tsu, Japan; b Department of Advanced Diagnostic Imaging, Mie University Graduate School of Medicine, Tsu, Japan; c Department of Gastrointestinal and Pediatric Surgery, Mie University Hospital, Tsu, Japan; d Department of Radiology, Mie University Graduate School of Medicine, Tsu, Japan; e Regional Co-creation Deployment Center, Mie Regional Plan Co-creation Organization, Mie University, Tsu, Japan

**Keywords:** Esophageal cancer, radiation therapy, cardiovascular complications, myocardial perfusion, intensity-modulated radiation therapy, proton beam therapy

## Abstract

**Background:**

Assessment of cardiac disease before cancer therapy is crucial, as advancements in cancer treatment have led to prolonged survival and an increase in cardiovascular complications. Specifically, esophageal cancer and heart disease share common risk factors, such as smoking and obesity. Radiation therapy (RT) for esophageal cancer is associated with elevated cardiac radiation exposure. This study aimed to assess the prevalence of coronary artery disease (CAD) in patients with esophageal cancer who were eligible for RT.

**Methods:**

We examined the prevalence of coronary artery stenosis, abnormal myocardial perfusion, and late enhancement using pre-RT cardiac computed tomography (CT) data of 41 patients with thoracic esophageal cancer who were referred for RT between January 2017 and June 2023 and had no history of ischemic heart disease.

**Results:**

The median age of the 41 patients was 71 years, with 40 patients being male. Cardiac CT identified significant coronary stenosis (≥50% luminal narrowing) in 18 patients (44%), among whom 9 (50%) had severe stenosis, multivessel disease, or myocardial ischemia. Significant stenosis was most frequently observed in the left anterior descending artery (16/18). Late enhancement, indicating myocardial infarction, was observed in seven patients (17%).

**Interpretation:**

Patients with esophageal cancer without a history of ischemic heart disease had a high prevalence (44%) of CAD, with half of them having severe stenosis, multivessel disease, or myocardial ischemia. Given the high prevalence of coronary stenosis, pre-treatment cardiac evaluation is crucial for patients with esophageal cancer. Incorporating cardiac CT findings into radiotherapy planning is recommended to optimize patient care.

## Graphical abstract

**Figure:**
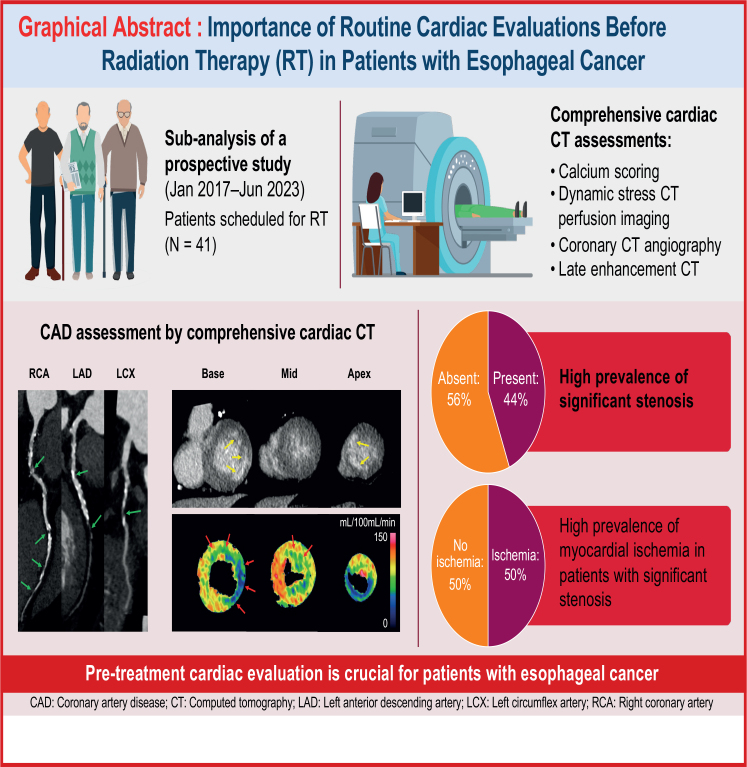

## Introduction

The field of cancer therapy has experienced remarkable advancements over the years, significantly improving patient survival rates across diverse cancer types. Radiation therapy (RT), in particular, has emerged as a pivotal component of cancer treatment, with techniques such as Intensity-Modulated Radiation Therapy (IMRT) and Proton beam therapy (PBT) enabling precise targeting of cancer cells while sparing surrounding healthy tissue [1–6]. However, the extension of life expectancy in cancer patients has also highlighted the long-term consequences of cancer therapies, particularly the increased risk of cardiovascular complications.

The heart’s vulnerability to radiation is well documented, with a dose–response relationship indicating that higher radiation doses to the heart increase the risk of various cardiac complications [7–10]. These risks are often delayed, with a latent period during which patients may appear unaffected, only to experience serious cardiac events years after treatment. In breast cancer, cardiac-related deaths have been reported to increase after postoperative irradiation of the left side of the breast compared to the right side [11]. This difference is likely due to the heart’s proximity to the radiation field on the left side, increasing the heart’s exposure to radiation and, consequently, the risk of long-term cardiac complications. In dose-comparison studies of esophageal and lung cancers, radiotherapy-induced cardiac injury has been suggested as a possible cause of poor performance in the high-dose group [12, 13].

Smoking and obesity are the most significant and well-established contributors to the development of esophageal cancer and coronary artery disease (CAD) [14, 15]. RT for esophageal cancer has been reported to be associated with a particularly high incidence of adverse cardiovascular events and cardiac-related deaths [16, 17]. Because RT for esophageal cancer often involves high doses of radiation to the heart, assessing the presence and severity of preexisting CAD before RT may contribute to improving the long-term outcomes of patients. The relationship between CAD and surgical complications is also an important consideration in esophageal cancer treatment. Patients with significant CAD might be at increased risk of perioperative cardiac events [18]. These risks are further compounded when surgery follows preoperative RT, as RT-induced cardiac injury can exacerbate existing coronary artery lesions and compromise myocardial function.

The concurrent prevalence of cardiovascular disease in patients with cancer, particularly esophageal cancer, poses critical challenges for clinical management, affecting treatment choices, potential cardiotoxic effects of treatments, and overall patient prognosis. Despite its importance, there remains a gap in comprehensive data on the prevalence of cardiovascular diseases in this patient group. This study aimed to clarify the prevalence of CAD among patients with esophageal cancer eligible for RT, using cardiac computed tomography (CT) to assess coronary artery stenosis, myocardial ischemia, and infarction before treatment.

## Material and methods

### Study design

**Figure 1:**
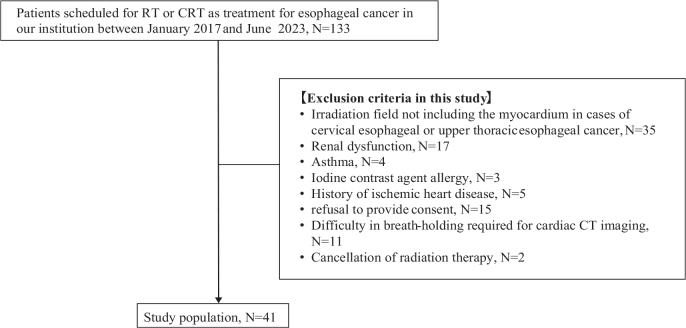
Study flowchart. The figure shows patient selection flowchart. Between January 2017 and June 2023, 133 patients scheduled for RT or CRT as treatment for esophageal cancer at our institution were considered for this clinical study. Of them, 90 patients were excluded based on the following ineligibility criteria: 1) irradiation fields not including the myocardium (*n* = 35); 2) renal dysfunction preventing the use of contrast agents (*n* = 17); 3) asthma (*n* = 4) or allergy to iodine contrast (*n* = 3); 4) history of ischemic heart disease (*n* = 5); 5) refusal to provide consent (*n* = 15); 6) difficulty in breath-holding for cardiac CT imaging (*n* = 11); and 7) cancellation of RT (*n* = 2). Finally, 41 patients were included for further analysis. RT: radiation therapy; CRT: chemoradiotherapy; CT: computed tomography.

This was a substudy of a prospective study in which comprehensive cardiac CT was performed before and after irradiation in patients undergoing irradiation for thoracic tumors (breast cancer, esophageal cancer, lung cancer, and lymphoma) to evaluate the presence of cardiac disease. The early results of this prospective study have already been published [19]. Between January 2017 and June 2023, 133 patients scheduled for RT or chemoradiotherapy (CRT) for esophageal cancer at our institution were considered for inclusion in this clinical study (Figure 1). Of them, 90 patients were excluded based on the following ineligibility criteria: (1) irradiation fields not including the myocardium (*n* = 35); (2) renal dysfunction preventing the use of contrast agents (*n* = 17); (3) asthma (due to the potential risks associated with iodine-based contrast agents and adenosine triphosphate) (*n* = 4) or allergy to iodine contrast (*n* = 3); (4) history of ischemic heart disease (*n* = 5); (5) refusal to provide consent (*n* = 15); (6) difficulty in breath-holding for cardiac CT imaging (*n* = 11); and (7) cancellation of RT (*n* = 2). Finally, 41 patients were included for further analysis. RT for these patients was planned for definitive, or curative, treatment. Of these, 31 underwent echocardiography, as this was a retrospective analysis based on available data. The Institutional Review Board approved this sub-study and waived the requirement for additional informed consent.

Image aquisition and Image analysis is shown in Supplementary materials.

### Statistical analysis

Continuous variables were expressed as the mean with standard deviation and, if necessary, the 95% confidence interval (CI). Parametric continuous variables were analyzed using the Student’s *t*-test. Non-parametric continuous variables were compared using the Mann–Whitney U test. Categorical variables were presented as numbers (percentages). The distribution of tumor stages was compared between patients with and without significant stenosis on coronary computed tomography angiography (CCTA) using the chi-square test. All statistical analyses were performed using SPSS version 23.0 (SPSS, Inc., Chicago, Illinois). Statistical significance was set at *p* < 0.05.

## Results

### Patient characteristics

Patient characteristics are shown in Table 1. The patient cohort consisted predominantly of males (97.6%, *n* = 40) with a median age of 71 years (range 49–86). The mean body mass index (BMI) was 20.69 ± 3.15 kg/m². Pathologically, 95.1% (*n* = 39) of the patients had squamous cell carcinoma and 4.9% (*n* = 2) had neuroendocrine carcinoma. Esophagogastroduodenoscopy, CT, and/or positron emission tomography (PET)/CT were used to stage esophageal tumors according to the 8th edition of the American Joint Committee on Cancer the primary tumor, lymph node and metastasis (TNM) classification, with disease staging showing 24.4% (*n* = 10) at stage I, 12.2% (*n* = 5) at stage II, 39.0% (*n* = 16) at stage III, and 24.4% (*n* = 10) at stage IV. The median radiation dose for esophageal cancer was 60 Gy (range 41.4–66). Among the patients, 29.3% (*n* = 12) received RT alone, while CRT was administered to 70.8% (*n* = 29). Treatment regimens included various chemotherapy combinations, with 46.3% (*n* = 19) receiving 5-Fluorouracil and Cisplatin. Out of the 41 patients, 9 (22%) underwent surgery after RT.

**Table 1:** Patients’ characteristics.

Variables	Value *n* = 41
**Age, median (range)**	71 (49–86) years
Sex	
Male	97.6% (*n* = 40)
**Body mass index (BMI), mean ± SD**	20.69 ± 3.15 kg/m2
**Pathology**	
Squamous cell carcinoma	95.1% (*n* = 39)
Neuroendocrine carcinoma	4.9% (*n* = 2)
**Stage (by the 8th UICC TNM classification)**	
I	24.4% (*n* = 10)
II	12.2% (*n* = 5)
III	39.0% (*n* = 16)
IV	24.4% (*n* = 10)
**TNM classification**	
T 1:2:3:4	11:5:19:6
N 0:1:2	17:13:10:1
M 0:1	37:4
**Radiation therapy**	
Radiation dose for the esophageal cancers, median (range)	60 (41.4–66) Gy
RT alone	29.3% (*n* = 12)
CRT	70.8% (*n* = 29)
CCRT	48.8% (*n* = 20)
NACRT	22.0% (*n* = 9)
**Combined with chemotherapy**	
None	29.3% (*n* = 12)
5-Fluorouracil and Cisplatin	46.3% (*n* = 19)
Leucovorin, Fluorouracil, and Oxaliplatin	9.8% (*n* = 4)
Cisplatin alone	7.3% (*n* = 3)
Carboplatin alone	2.4% (*n* = 1)
Cisplatin and Etoposide	2.4% (*n* = 1)
Carboplatin and Etoposide	2.4% (*n* = 1)
**Coronary risk factors**	
Smoking history	82.9% (*n* = 34)
Smoking history with Brinkman index ≥400	65.9% (*n* = 27)
Hypertension	43.9% (*n* = 18)
Hyperlipidemia	7.3% (*n* = 3)
Diabetes	9.8% (*n* = 4)
Obesity (BMI ≥ 25kg/m2)	12.2% (*n* = 5)
Pretest probability of CAD, mean ± SD	26.3% ± 14.1%
**Symptom**	
Chest pain	0% (*n* = 0)
Dyspnea	0% (*n* = 0)
**Blood test (*n* = 30)**	
BNP (pg/mL)	26.7 (13.6–51.3)
BNP ≥ 18.4pg/mL	48.8% (*n* = 20)

Values are presented as percentages (number of patients) unless indicated otherwise. CRT: chemoradiotherapy; CCRT: concurrent chemoradiotherapy; NACRT: neoadjuvant chemoradiotherapy; CAD: coronary artery disease; BNP: brain natriuretic peptide.

Coronary risk factors were prevalent, with a significant smoking history in 82.9% (*n* = 34) and hypertension in 43.9% (*n* = 18) of the patients. Pretest probability of obstructive CAD in the patients was 26.3 ± 14.1% by clinical model according to the CAD Consortium [20]. None of the patients presented with chest pain or dyspnea. Blood tests for 30 patients revealed that 48.8% (*n* = 20) had BNP levels equal to or greater than 18.4 pg/mL.

### Results of cardiac CT and echocardiography

**Figure 2:**
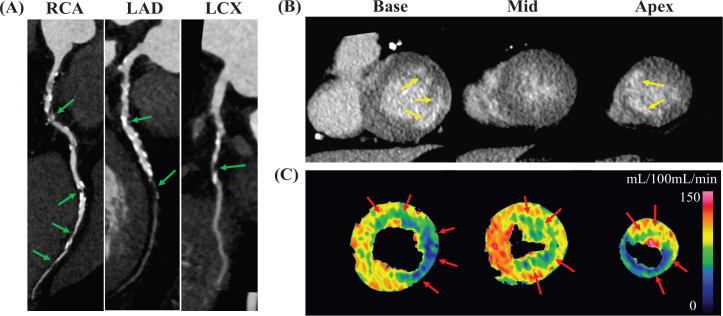
Representative case of CAD assessment by comprehensive cardiac CT. The figure shows results of CAD assessment in a 70-year-old male with esophageal cancer using comprehensive cardiac CT, including (A) CCTA images (curved multiplanar reconstruction), (B) LECT images (short axis), and (C) dynamic CTP maps (short axis). On the CCTA images, significant stenoses (green arrows) in all three coronary arteries were observed. The LECT images revealed enhancement areas showing infarction (yellow arrows) in the basal lateral and apical septal walls. The dynamic CTP maps demonstrated perfusion abnormalities suggestive of myocardial ischemia in myocardial areas that did not display infarction. CAD: coronary artery disease; CT: computed tomography; CCTA: coronary computed tomography angiography; RCA: right coronary artery; LAD: left anterior descending artery; LCX: left circumflex artery; LECT: late enhancement computed tomography; CTP: computed tomography perfusion.

**Figure 3:**
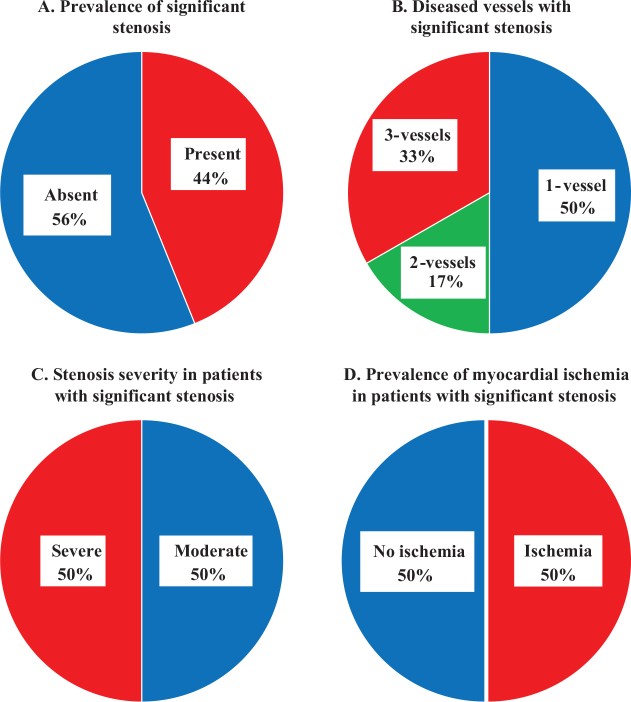
Distribution and severity of significant stenosis and its association with myocardial ischemia. The figure presents data on the prevalence and characteristics of significant stenosis in patients: (A) significant stenosis was present in 44% (18/41) of the patients; (B) of the patients with significant stenosis, 50% (9/18) had 1-vessel disease, 17% (3/18) had 2-vessel disease, and 33% (6/18) had 3-vessel disease; (C) significant stenosis was categorized as moderate in 50% (9/18) of patients and severe in 50% (9/18) based on the maximal stenosis severity when multiple significant stenoses were present; and (D) myocardial ischemia was observed in 50% (9/18) of the patients. Central Illustration: [Title. Caption.]

Cardiac CT findings of the patient cohort are presented in Table 2. Among the 41 cases, 14 cases (34%) showed no calcification, and among them, 12 cases (86%) were found to have no significant stenosis according to CAD-RADS. A representative case is shown in Figure 2. Coronary artery involvement was observed in 44.0% (*n* = 18) of patients (Figure 3), with one affected artery in 22.0% (*n* = 9) of the patients, two affected arteries in 7.3% (*n* = 3), and three affected arteries in 14.6% (*n* = 6). The left anterior descending artery (LAD) was the most frequently involved artery, affected in 39% (*n* = 16) of cases. Abnormal perfusion on CTP was observed in 39.0% (*n* = 16) of the participants, with an ischemic pattern observed in 21.9% (*n* = 9) and a non-ischemic pattern in 17.1% (*n* = 7). Late enhancement on LECT was detected in 19.5% (*n* = 8) of the participants, predominantly with an ischemic pattern (17.1% [*n* = 7]) and to a lesser extent with a non-ischemic pattern (2.4% [*n* = 1]).

**Table 2:** Findings of cardiac CT.

Variables	Value *n* = 41
**Calcium score**	
0	34.1% (*n* = 14)
1–399	31.7% (*n* = 13)
≥ 400	34.1% (*n* = 14)
**CAD-RADS (CCTA)**	
0	29.3% (*n* = 12)
1	4.9% (*n* = 2)
2	22.0% (*n* = 9)
3	22.0% (*n* = 9)
4A	9.8% (*n* = 4)
4B	9.8% (*n* = 4)
5	2.4% (*n* = 1)
**Diseased coronary artery with significant stenosis (CCTA)**	
One coronary artery	22.0% (*n* = 9)
Two coronary arteries	7.3% (*n* = 3)
Three coronary arteries	14.6% (*n* = 6)
Left main coronary trunk	2.4% (*n* = 1)
Left anterior descending artery	39% (*n* = 16)
Left circumflex artery	19.5% (*n* = 8)
Right coronary artery	19.5% (*n* = 8)
**Abnormal perfusion on CTP**	39.0% (*n* = 16)
Macrovascular pattern	21.9% (*n* = 9)
Microvascular pattern	17.1% (*n* = 7)
**Late enhancement on LECT**	19.5% (*n* = 8)
Ischemic pattern (infarction)	17.1% (*n* = 7)
Non-ischemic pattern	2.4% (*n* = 1)

CAD-RADS: coronary artery disease reporting and data system; CCTA: coronary computed tomography angiography

Supplementary Table 1 compares significant stenosis in patients across various stages and TNM classifications. Overall, no significant differences were observed in the presence of significant stenosis.

The radiation dose for cardiac CT is shown in Supplementary Table 2.

The echocardiogram findings for 31 patients revealed that 6 patients (19%) exhibited abnormalities, including LV systolic or diastolic dysfunction. Of the 31 patients who underwent echocardiography, 13 (42%) showed abnormalities on cardiac CT. Among the 31 patients who underwent echocardiography, the following abnormalities were detected on cardiac CT: 13 (42%) had significant stenosis; 7 (23%) or 6 (19%) had perfusion abnormalities with macrovascular or microvascular patterns, respectively; 6 (19%) had infarction; and 1 (3%) had non-ischemic late enhancement (Supplementary Table 3).

## Discussion

### Radiation-induced heart disease

RT is crucial for managing esophageal cancer, whether as definitive therapy, adjunct to surgery, or for palliative care. However, RT poses a significant risk of late cardiopulmonary toxicity, including radiation pneumonitis and pericarditis, affecting up to 30% of patients aged > 75 [17]. Studies, including those on breast cancer, have demonstrated that an increased radiation dose to the heart correlated with higher rates of coronary events, implying the importance of minimizing cardiac exposure, especially with elevated radiation doses. Techniques such as IMRT and PBT may improve local control and curative potential while minimizing complications [1–6], with PBT showing particular promise for reducing Grade 3 cardiac events in advanced stages without compromising overall or progression-free survival [6].

### Cancer and cardiovascular disease

The concurrent prevalence of cardiovascular disease in patients with cancer, particularly esophageal cancer, poses critical challenges for clinical management, affecting treatment choices, potential cardiotoxic effects of treatments, and overall patient prognosis. Despite its importance, there remains a gap in comprehensive data on the prevalence of cardiovascular diseases in this patient group. In this study, cardiac CT scans prior to RT in patients with esophageal cancer revealed significant coronary artery stenosis in 44% (18/41) of patients, with a concerning 33% (6/18) of these patients exhibiting critical three-vessel disease. Abnormal myocardial blood flow was observed in 39% (16/41) of patients, while myocardial infarctions or fibrosis were identified in 20% (8/41) of the patients. These figures indicate a high baseline risk of cardiac complications before RT introduction. These findings align with broader research, such as that by Muhummad et al. [21], who showed a 22% incidence of significant stenosis among patients with various cancer types who underwent cardiac CT, emphasizing that patients with esophageal cancer have a markedly higher prevalence of severe coronary stenosis. This pretreatment cardiac vulnerability suggests a potential severity of radiation-induced cardiac injury in esophageal cancer, highlighting the importance of a thorough cardiac assessment before initiating RT to tailor safer treatment plans.

### The role of cardiac CT for esophageal cancer

CCTA provides detailed visualization of the coronary arteries, allowing for the identification of significant stenoses. In our study, significant coronary stenosis on CCTA was detected in 44% of the patients, with 89% of these patients exhibiting lesions in the LAD. Proximal LAD lesions are strong prognostic indicators of adverse cardiac events in the general population. Significant stenosis in the proximal LAD is associated with increased mortality within 1–3 years, independent of other coronary artery lesions [24]. This implies the critical importance of detecting LAD lesions before RT in patients with esophageal cancer, as radiation exposure can exacerbate existing coronary artery stenosis and lead to severe cardiac complications. Techniques, such as IMRT and PBT, can be strategically employed to spare the LAD from excessive radiation doses. While CCTA offers valuable insights into coronary anatomy, comprehensive cardiac CT – including CTP and LECT – offers additional information on myocardial ischemia and infarction/fibrosis. Abnormal MBF, indicative of myocardial ischemia, and late enhancement, suggestive of infarction/fibrosis, have been identified as independent predictors of major adverse cardiac events, adding prognostic value beyond that provided by CCTA alone [22, 23]. Detection of myocardial ischemia and infarction/fibrosis can further refine RT planning by identifying regions of the myocardium that are already compromised and may be more susceptible to radiation-induced damage. This comprehensive approach enables the development of a more personalized and safer RT plan, potentially reducing the risk of various radiation-induced cardiac complications, including coronary artery injury, myocardial injury, arrhythmias, heart failure, and pericarditis.

However, comprehensive cardiac CT may not be universally accessible or routinely used in all clinical settings owing to resource limitations. In such cases, alternative strategies should be considered. CCTA alone can still offer significant insights into the burden and location of atherosclerotic disease, particularly in the LAD, which is critical for RT planning. If CTP is unavailable, myocardial perfusion can be evaluated using modalities, such as single-photon emission computed tomography or cardiac magnetic resonance imaging. The decision regarding which specific imaging components to include should be guided by the clinical context and availability of resources. However, when comprehensive cardiac CT is available, it should be considered more frequently, particularly in high-risk patients such as those with multiple cardiovascular risk factors because, its ability to provide detailed assessments of both coronary anatomy and myocardial conditions in a single examination makes it an invaluable tool for integrating cardiac information into radiotherapy planning.

### Planning CT and cardiac CT

Planning CT, though routinely acquired for radiotherapy preparation, has limited utility in identifying CAD due to its lack of contrast enhancement, lower image resolution, and the absence of Electrocardiogram (ECG) gating. Without ECG gating, planning CT cannot reliably capture the coronary arteries in their optimal phase, leading to motion artifacts and reduced diagnostic accuracy. While it may incidentally reveal coronary calcifications, planning CT cannot adequately assess non-calcified plaques or the functional significance of stenoses. In contrast, cardiac CT provides ECG-gated, high-resolution imaging with contrast enhancement, enabling precise evaluation of coronary anatomy and the detection of hemodynamically significant CAD. While enhancing planning CT protocols could offer some potential for CAD evaluation, its current limitations make it insufficient as a standalone modality. Therefore, dedicated cardiac CT remains essential for accurate CAD assessment, leading to optimized radiotherapy planning. Interestingly, in this study, 12 out of 14 cases (86%) without coronary artery calcification had no significant coronary artery stenosis. This suggests that performing cardiac CT selectively, only in patients with coronary artery calcification detected on planning CT, could be a resource-efficient approach. While planning CT has limitations, its ability to incidentally detect coronary calcifications may serve as a useful triage tool to identify patients who would benefit most from further cardiac evaluation using CCTA or comprehensive cardiac CT.

### Echocardiography and cardiac CT

The study indicated the limitations of echocardiography alone in identifying significant cardiac abnormalities. In our cohort, echocardiography detected abnormalities in only 19% of patients, whereas cardiac CT revealed a much higher prevalence of significant coronary artery stenosis (44%), myocardial ischemia and infarction/fibrosis. This disparity suggests the importance of incorporating advanced imaging modalities, such as CCTA and CTP, in the pre-treatment evaluation of esophageal cancer patients to uncover clinically relevant cardiac conditions that may influence treatment planning and outcomes.

### Collaborative approach to patient care

The insights gained from comprehensive cardiac CT examination empower a more informed and collaborative approach to patient care. With a detailed assessment of the patient’s cardiac health, the medical team, including cardiologists, radiation oncologists, and other specialists, can engage in multidisciplinary discussions to create a holistic treatment strategy that addresses both cancer and any preexisting or potential cardiac conditions. This approach not only ensures a personalized treatment plan that is finely tuned to the specific needs and risks of each patient but also promotes an environment of shared decision-making. Patients benefit from a better understanding of their health status and treatment options, leading to more informed care choices. When CAD is detected during pre-treatment evaluation, a comprehensive management strategy is essential to address both the oncologic and cardiovascular needs of the patient. This involves collaboration among cardiologists, radiation oncologists, and, where applicable, surgeons, to balance the immediate need for cancer treatment with the potential risks posed by cardiac disease. For patients with mild to moderate stenosis, medical optimization is generally sufficient, alongside close cardiac monitoring during cancer treatment. In cases of severe stenosis, revascularization procedures might be necessary prior to initiating cancer therapy. Radiation oncologists should integrate cardiac CT findings into radiotherapy planning, employing advanced techniques such as IMRT or PBT to minimize radiation exposure to the heart. For patients undergoing surgery, preoperative identification of CAD allows for tailored perioperative cardiac management. This multidisciplinary approach ensures that the dual goals of effective cancer treatment and cardiovascular safety are met, potentially improving patient outcomes.

Ultimately, this comprehensive cardiac assessment underlines the importance of an integrated treatment paradigm in oncology that harmonizes the objective of cancer eradication with the need for cardiovascular safety, thereby advancing the goal of improving patient outcomes in the field of cardio-oncology.

### Limitations

This study has several limitations. Firstly, the study’s findings were based on a relatively small cohort of 41 patients. This may limit the statistical power of our study and the generalizability of its findings to broader populations. Additionally, the demographics of our study cohort consisted predominantly of elderly male smokers, who reflected the population most affected by esophageal cancer. This also limits the generalizability of our findings to women and non-smoking populations, who may present with different cardiac risk profiles and disease prevalence. Future studies with larger, more diverse cohorts are needed to validate these results. Secondly, as a sub-study of a previously published prospective study conducted at a single center, the findings might reflect the specific patient population and practices of that institution, potentially limiting their applicability to other settings. Thirdly, the study primarily focused on pre-RT cardiac CT data without detailed information on long-term cardiovascular outcomes post-RT. This limitation restricts the ability to fully understand the impact of identified cardiac abnormalities on patient prognosis and survival. Fourthly, the study lacks a comparison group of individuals without cancer but with similar cardiovascular risk profiles. Fifthly, this study did not include detailed analyses of surgical effects. Future research should examine the interplay between CAD, RT heart doses, and surgical outcomes in esophageal cancer patients to better inform treatment strategies. Finally, due to the retrospective nature of the sub-analysis, not all patients underwent echocardiography, which may be related to selection bias; however, the prevalence of cardiac CT abnormalities in patients who underwent echocardiography did not differ significantly from that in the overall cohort. Despite these limitations, this study contributes significantly to the understanding of cardiovascular risks in patients with esophageal cancer and underlines the importance of integrating cardio-oncology into treatment planning.

## Conclusions

Patients with esophageal cancer and no history of ischemic heart disease exhibited a high prevalence of CAD at 44%, which is significantly higher than would be predicted based solely on traditional clinical risk factors. Notably, half of these patients had severe stenosis, multivessel disease, or myocardial ischemia. Considering the high prevalence of coronary stenosis, pre-treatment cardiac evaluation is crucial for patients with esophageal cancer. Incorporating cardiac CT findings into radiotherapy planning is recommended to optimize patient care. Furthermore, cardiac CT findings can help identify patients who would benefit from IMRT or PBT, thereby guiding the selection of the most appropriate radiotherapy technique. These insights underscore the necessity for an integrated approach to cancer treatment that balances oncological efficacy with cardiovascular safety.

## Supplementary Material

Cardiac CT reveals high prevalence of coronary artery disease in esophageal cancer eligible for radiotherapy

## Data Availability

Available if reasonably requested.

## References

[1] Wang J, Wei C, Tucker SL, Myles B, Palmer M, Hofstetter WL, et al. Predictors of postoperative complications after trimodality therapy for esophageal cancer. Int J Radiat Oncol Biol Phys. 2013;86(5):885–91. 10.1016/j.ijrobp.2013.04.006 23845841 PMC3786201

[2] Lin SH, Zhang N, Godby J, Wang J, Marsh GD, Liao Z, et al. Radiation modality use and cardiopulmonary mortality risk in elderly patients with esophageal cancer. Cancer. 2016;122(6):917–28. 10.1002/cncr.29857 26716915 PMC6095128

[3] Lin SH, Wang L, Myles B, Thall PF, Hofstetter WL, Swisher SG, et al. Propensity score-based comparison of long-term outcomes with 3-dimensional conformal radiotherapy vs intensity-modulated radiotherapy for esophageal cancer. Int J Radiat Oncol Biol Phys. 2012;84(5):1078–85. 10.1016/j.ijrobp.2012.02.015 22867894 PMC3923623

[4] He L, Chapple A, Liao Z, Komaki R, Thall PF, Lin SH. Bayesian regression analyses of radiation modality effects on pericardial and pleural effusion and survival in esophageal cancer. Radiother Oncol. 2016;121(1):70–4. 10.1016/j.radonc.2016.08.005 27562616 PMC5546146

[5] Xi M, Xu C, Liao Z, Chang JY, Gomez DR, Jeter M, et al. Comparative outcomes after definitive chemoradiotherapy using proton beam therapy versus intensity modulated radiation therapy for esophageal cancer: a retrospective, single-institutional analysis. Int J Radiat Oncol Biol Phys. 2017;99(3):667–76. 10.1016/j.ijrobp.2017.06.2450 29280461

[6] Wang X, Palaskas NL, Yusuf SW, Abe JI, Lopez-Mattei J, Banchs J, et al. Incidence and onset of severe cardiac events after radiotherapy for esophageal cancer. J Thorac Oncol. 2020;15(10):1682–90. 10.1016/j.jtho.2020.06.014 32599073 PMC9398884

[7] van den Bogaard VA, Ta BD, van der Schaaf A, Bouma AB, Middag AM, Bantema-Joppe EJ, et al. Validation and modification of a prediction model for acute cardiac events in patients with breast cancer treated with radiotherapy based on three-dimensional dose distributions to cardiac substructures. J Clin Oncol. 2017;35(11):1171–8. 10.1200/JCO.2016.69.8480 28095159 PMC5455600

[8] Banfill K, Giuliani M, Aznar M, Franks K, McWilliam A, Schmitt M, et al. Cardiac toxicity of thoracic radiotherapy: existing evidence and future directions. J Thorac Oncol. 2021;16(2):216–27. 10.1016/j.jtho.2020.11.002 33278607 PMC7870458

[9] Frandsen J, Boothe D, Gaffney DK, Wilson BD, Lloyd S. Increased risk of death due to heart disease after radiotherapy for esophageal cancer. J Gastrointest Oncol. 2015;6(5):516–23. 26487946 10.3978/j.issn.2078-6891.2015.040 PMC4570919

[10] Pao TH, Chang WL, Chiang NJ, Lin CY, Lai WW, Tseng YL, et al. Pericardial effusion after definitive concurrent chemotherapy and intensity modulated radiotherapy for esophageal cancer. Radiat Oncol. 2020;15(1):48. 10.1186/s13014-020-01498-3 32103779 PMC7045635

[11] Darby SC, Ewertz M, McGale P, Bennet AM, Blom-Goldman U, Bronnum D, et al. Risk of ischemic heart disease in women after radiotherapy for breast cancer. N Engl J Med. 2013;368(11):987–98. 10.1056/NEJMoa1209825 23484825

[12] Minsky BD, Pajak TF, Ginsberg RJ, Pisansky TM, Martenson J, Komaki R, et al. INT 0123 (Radiation Therapy Oncology Group 94-05) phase III trial of combined-modality therapy for esophageal cancer: high-dose versus standard-dose radiation therapy. J Clin Oncol. 2002;20(5):1167–74. 10.1200/JCO.2002.20.5.1167 11870157

[13] Bradley JD, Paulus R, Komaki R, Masters G, Blumenschein G, Schild S, et al. Standard-dose versus high-dose conformal radiotherapy with concurrent and consolidation carboplatin plus paclitaxel with or without cetuximab for patients with stage IIIA or IIIB non-small-cell lung cancer (RTOG 0617): a randomised, two-by-two factorial phase 3 study. Lancet Oncol. 2015;16(2):187–99. 10.1016/S1470-2045(14)71207-0 25601342 PMC4419359

[14] Kamimura D, Cain LR, Mentz RJ, White WB, Blaha MJ, DeFilippis AP, et al. Cigarette smoking and incident heart failure: insights from the Jackson heart study. Circulation. 2018;137(24):2572–82. 10.1161/CIRCULATIONAHA.117.031912 29661945 PMC6085757

[15] Guenancia C, Lefebvre A, Cardinale D, Yu AF, Ladoire S, Ghiringhelli F, et al. Obesity as a risk factor for anthracyclines and trastuzumab cardiotoxicity in breast cancer: a systematic review and meta-analysis. J Clin Oncol. 2016;34(26):3157–65. 10.1200/JCO.2016.67.4846 27458291 PMC5569689

[16] Ishikura S, Nihei K, Ohtsu A, Boku N, Hironaka S, Mera K, et al. Long-term toxicity after definitive chemoradiotherapy for squamous cell carcinoma of the thoracic esophagus. J Clin Oncol. 2003;21(14):2697–702. 10.1200/JCO.2003.03.055 12860946

[17] Morota M, Gomi K, Kozuka T, Chin K, Matsuura M, Oguchi M, et al. Late toxicity after definitive concurrent chemoradiotherapy for thoracic esophageal carcinoma. Int J Radiat Oncol Biol Phys. 2009;75(1):122–8. 10.1016/j.ijrobp.2008.10.075 19327900

[18] Wright CD, Kucharczuk JC, O’Brien SM, Grab JD, Allen MS, Society of Thoracic Surgeons General Thoracic Surgery D. Predictors of major morbidity and mortality after esophagectomy for esophageal cancer: a Society of Thoracic Surgeons General Thoracic Surgery Database risk adjustment model. J Thorac Cardiovasc Surg. 2009;137(3):587–95; discussion 96. 10.1016/j.jtcvs.2008.11.042 19258071

[19] Takada A, Ichikawa Y, Nakamura S, Toyomasu Y, Kawamura T, Nanpei Y, et al. Preliminary results of reduced myocardial blood flow in the subacute phase after radiation therapy for thoracic esophageal cancer: a quantitative analysis with stress dynamic myocardial computed tomography perfusion imaging. Radiother Oncol. 2022;177:191–6. 10.1016/j.radonc.2022.11.002 36372209

[20] Bittencourt MS, Hulten E, Polonsky TS, Hoffman U, Nasir K, Abbara S, et al. European Society of cardiology-recommended coronary artery disease consortium pretest probability scores more accurately predict obstructive coronary disease and cardiovascular events than the diamond and forrester score: the partners registry. Circulation. 2016;134(3):201–11. 10.1161/CIRCULATIONAHA.116.023396 27413052

[21] Nazir MS, Murphy T, Poku N, Wheen P, Nowbar AN, Andres MS, et al. Clinical utility and prognostic value of coronary computed tomography angiography in patients with cancer. Am J Cardiol. 2023;207:448–54. 10.1016/j.amjcard.2023.08.121 37797552

[22] Nakamura S, Kitagawa K, Goto Y, Omori T, Kurita T, Yamada A, et al. Incremental prognostic value of myocardial blood flow quantified with stress dynamic computed tomography perfusion imaging. JACC Cardiovasc Imaging. 2019;12(7 Pt 2):1379–87. 10.1016/j.jcmg.2018.05.021 30031698

[23] Nakamura S, Kitagawa K, Goto Y, Takafuji M, Nakamori S, Kurita T, et al. Prognostic value of stress dynamic computed tomography perfusion with computed tomography delayed enhancement. JACC Cardiovasc Imaging. 2020;13(8):1721–34. 10.1016/j.jcmg.2019.12.017 32061554

[24] Klein LW, Weintraub WS, Agarwal JB, Schneider RM, Seelaus PA, Katz RI, et al. Prognostic significance of severe narrowing of the proximal portion of the left anterior descending coronary artery. American Journal of Cardiology. 1986;58(1):42-6. 10.1016/0002-9149(86)90238-9 3728330

