# Supplementary material for: Cardiac CT reveals high prevalence of coronary artery disease in esophageal cancer eligible for radiotherapy
Source: Acta Oncol. 2025 Feb 3;64:42563. doi: 10.2340/1651-226X.2025.42563 (PMC11811530; doi:10.2340/1651-226X.2025.42563)
Supplement: Cardiac CT reveals high prevalence of coronary artery disease in esophageal cancer eligible for radiotherapy [file AO-64-42563-s1.pdf]

Supplementary material has been published as submitted. It has not been copyedited, or typeset by Acta Oncologica

Supplementary material

Image acquisition

All cardiac CT images were acquired using a third-generation dual-source CT scanner (SOMATOM Force; Siemens Healthcare, Erlangen, Germany). Comprehensive cardiac CT included calcium scoring, dynamic stress CT perfusion imaging (CTP), coronary CT angiography (CCTA), and late-enhancement CT (LECT). Detailed CT protocols have been described previously (1). Briefly, following calcium scoring, dynamic stress CTP was initiated more than three minutes after administering adenosine triphosphate at a rate of 0.16 mg/kg/min by injecting 40 mL of iopamidol. Dynamic datasets were acquired in the end-systolic phase for 30 seconds via electrocardiogram-triggered axial scan mode repeated at two alternating table positions (i.e., “shuttle mode”). Ten minutes after dynamic stress CTP, standard prospective CCTA was performed at rest by a bolus injection of 26-mg-iodine/kg/s of iopamidol over 12 seconds with the coronary arteries dilated with nitrate. Heart rate was controlled before CCTA with an intravenous injection of a beta-blocker, if necessary. A LECT scan was performed 5 minutes after coronary CTA.

References

1. Takada A, Ichikawa Y, Nakamura S et al. Preliminary results of reduced myocardial blood flow in the subacute phase after radiation therapy for thoracic esophageal cancer: A quantitative analysis with stress dynamic myocardial computed tomography perfusion imaging. Radiotherapy and Oncology 2022;177:191-196.

Image analysis

CCTA images were evaluated by two experienced radiologists in a joint reading. The presence and severity of coronary artery stenosis on CCTA was assessed using the Coronary Artery Disease-Reporting and Data System (CAD-RADS) classification (1): 0 (0%); 1 (1–24%), 2 (25–49%), 3 (50–69%), 4A (70–99% in 1 to 2 vessels), 4B (70–99% in 3 vessels or ≥50% left main), or 5 (100%). Significant stenosis was defined as ≥50% luminal narrowing in the coronary arteries.

From the CTP image data, myocardial blood flow (MBF) maps were generated semiautomatically using a parametric deconvolution method with commercially available perfusion analysis software (Syngo VPCT body, Siemens Healthcare). Two diagnostic radiologists, experienced in cardiac CT and unaware of the dosimetry results, visually evaluated the CTP and LECT images side-by-side to identify the presence of MBF abnormalities or late enhancement in each myocardial segment. Perfusion abnormalities were identified on the MBF map in two scenarios: when decreased MBF was observed in segments without late enhancement on LECT images or when the area of decreased MBF was larger than the area of late enhancement. Perfusion abnormalities were classified as macrovascular if attributable to coronary artery stenosis based on coronary CT findings and as microvascular if not explained by coronary artery stenosis. Subendocardial or transmural late enhancement in a coronary distribution was considered an ischemic pattern (infarction), while patchy or diffuse enhancement in the midwall or subepicardium with sparing of the subendocardium was considered a non-ischemic pattern (2).

References

1. Cury RC, Abbara S, Achenbach S et al. CAD-RADSTM coronary artery disease–reporting and data system. An expert consensus document of the Society of Cardiovascular Computed Tomography (SCCT), the American College of Radiology (ACR) and the North American Society for Cardiovascular Imaging (NASCI). Endorsed by the American College of Cardiology. Journal of cardiovascular computed tomography 2016;10(4):269-281.
2. McCrohon JA, Moon JC, Prasad SK et al. Differentiation of heart failure related to dilated cardiomyopathy and coronary artery disease using gadolinium-enhanced cardiovascular magnetic resonance. Circulation 2003;108:54-9.

Supplementary table 1.

Distribution of tumor stage in patients with or without significant stenosis

**Variables**	**Significant stenosis**		**P value**
	**Present (n=18)**	**Absent (n=23)**	
Stage			
I	33.3% (n=6)	17.4% (n=4)	
II	16.7% (n=3)	8.7% (n=2)	P=0.256
III	22.2% (n=4)	52.2% (n=12)	
IV	27.8% (n=5)	21.7% (n=5)	
TNM classification			
T1	33.3% (n=6)	21.7% (n=5)	
T2	16.7% (n=3)	8.7% (n=2)	P=0.197
T3	27.8% (n=5)	60.9% (n=14)	
T4	22.2% (n=4)	8.7% (n=2)	
N0	50.0% (n=9)	34.8% (n=8)	
N1	22.2% (n=4)	39.1% (n=9)	P=0.492
N2	27.8% (n=5)	21.7% (n=5)	
N3	0% (n=0)	4.3% (n=1)	
M0	88.9% (n=16)	91.3% (n=21)	P=0.798
M1	11.1% (n=2)	8.7% (n=2)	

Supplementary table 2. Radiation dose of Cardiac CT

Variables	Value
**Calcium score**	
Dose length product: mean ± SD	11±3 mGy･cm
Effective radiation dose: mean ± SD	0.2±0.1 mSv
**CCTA**	
Dose length product: mean ± SD	148±128 mGy･cm
Effective radiation dose: mean ± SD	2.1±1.8 mSv
**Dynamic CTP**	
Dose length product: mean ± SD	211±58 mGy･cm
Effective radiation dose: mean ± SD	3.0±0.8 mSv
**LECT**	
Dose length product: mean ± SD	142±24 mGy･cm
Effective radiation dose: mean ± SD	2.0±0.3 mSv

CCTA, coronary computed tomography angiography; CTP, computed tomography perfusion; LECT, late enhancement computed tomography.

Supplementary table 3.

Findings of echocardiogram (n=31)

Variables	Value
LVEF (Parasternal long) (%)	67.7±10.5
LVEF 40–50%	3.2% (n=1)
LVEF <40%	3.2% (n=1)
LVMI (g/m^2)	87.1±26.0
LVEDV (ml)	96.7±38.7
LVESV (ml)	34.2±29.8
LAVI (ml/m^2)	32.5±18.2
LAVI >34ml/m^2	19.4% (n=6)
e’ (septal) (cm/s)	6.3±1.9
e’ (lateral) (cm/s)	9.0±2.3
e’ (septal) <7cm/s or e’ (lateral) <10cm/s	74.2% (n=23)
E/e’ (septal)	11.1±5.9
E/e’ (lateral)	7.7±3.0
E/e’ (average of lateral and septal e’)	9.3±4.4
E/e’ (average of lateral and septal e’) >14.0	6.5% (n=2)
LV diastolic dysfunction*	12.9% (n=4)

LV, left ventricle; LVEF, left ventricular ejection fraction; LVMI, left ventricular mass index; LVEDV, left ventricular end-diastolic volume; LVESV, left ventricular end-systolic volume; LAVI, left atrial volume index.

*LV diastolic dysfunction was defined as being positive for two or more of the following three variables: LAVI >34 ml/m^2, e’ (septal) <7 cm/s or e’ (lateral) <10 cm/s, and E/e’ (average of lateral and septal e’) >14.0.
